# 

**Published:** 1869-03

**Authors:** 


### Books and Pamphlets Received.

The Structural Lesions of the Skin; their Pathology and Treatment illustrated. By Howard F. Damon, A. M., M. D. Philadelphia: J. B. Lippincott & Co., 1869. Received through and for sale by Breed & Lent, Buffalo.

Chloroform, and a New Method of Administering it. By A. M. Rosebrugh, M. D., Surgeon to the Toronto Charitable Eye Dispensary.

Pathological Phenomena Generalized. By H. Backus, Montevallo, Ala.

The Life of the Trichina. By Rudolph Virchow, M. D., PH. D., Professor University of Berlin. Translated by Rufus King Brown, M. D.

Remarks on Dr. Sayre's Paper entitled "A New Operation for Artificial Hip-joint, in Bony Anchylosis." By Louis Bauer, M. D., of Brooklyn.

Enucleation of the Eyeball. By B. Joy Jeffries, A. M., M. D.

The Part taken by Nature and Time in the Cure of Diseases. By James F. Hibberd, M. D.

Quarterly Summary of the Transactions of the College of Physicians of Philadelphia, from December 5, 1866, to December 2, 1868, inclusive.

Medical Department, Georgetown College, 1868–69.

### American Medical Association,

*Office of Permanent Secretary.*

Wm. B. Atkinson, M. D.,

S. W. corner Broad and Pine streets, Philadelphia, Pa.

The Twentieth Annual Session will be held in New Orleans, May 4th, 1869, at 11 A. M.

Secretaries of all medical organizations are requested to forward lists of their del egates as soon as elected, to the Permanent Secretary.

Any respectable physician who may desire to attend, but cannot do so as a delegate, may be made a *member by invitation*, upon the recommendation of the Committee of Arrangements. W. B. Atkinson.

